# Supplementary material for: Acceptance and compliance with micronutrient powder (MNP) among children aged 6–23 months in northern Nigeria
Source: PLOS Glob Public Health. 2022 Oct 17;2(10):e0000961. doi: 10.1371/journal.pgph.0000961 (PMC10022258; doi:10.1371/journal.pgph.0000961)
Supplement: S1 Dataset — (ZIP) [file pgph.0000961.s008.zip › S8_Dataset/Direct Observations.zip!/Direct Observations/230615_K_J_SU_1_DO.pdf]

1.1.1 Instrument F –Direct Observation Form

<<Instrument F Direct Observation Form>>
**PI Name:** PragyaMathema,Stephen Kodish<br>**Study Title:** Formative research to develop an infant and young child feeding (IYCF) intervention with micronutrient powder (MNP) for children aged 6 – 23 months<br>**PI Version/Date:** Version1.0/January 24, 2015

Date: 23/06/2015 Location: Ruga

Data Collector: Samira Usman

Main goals of this direct observation are to answer the following questions:
1. In this household, what would be the best way(s) to give nutritional supplements to young children? Why?<br><br>2. In this household, describe the food sharing that you observe throughout the meal. Are there any predictable patterns?<br><br>3. In this household, who is favored and how is this favoritism expressed during meal preparation and eating?

Household Composition Table (Fill out as you do data collection)

INITIALS	SEX (M/F)	AGE (estimated)	Relationship to household	Description
MM	F	22yrs	Mother of the baby Daughter in-law	Tall, dark wearing green brown wrapper and a veil on her west, green head tie
BB	M	12 months	son	Naked baby with blue/white necklace
FF	F	25yrs	Daughter in-law	Tall fair wearing Ankara wrapper, green cap and head tie
SG	F	10yrs	Daughter	Blue skirt and black cap
MI	F	50yrs	Mother in-law	Tall fair wearing green and blue Ankara with white veil
FI	M	58yrs	Father in-law	Tall fair dark blue gown trouser shadda and brown cap
SB	M	7yrs	son	Fair boy wearing light blue shadda
TB	F	20yrs	Daughter in-law	Tall, dark wearing wrapper, short and black head tie
SD	F	16yrs	Daughter in-law	Average height, dark wearing wrapper

				and a veil head tie on the top
BB2	M	3yrs	Son his mother left the house	Naked baby
HI	M	32yrs	husband	Tall slim wearing trouser top and a white cap
H2	M	31yrs	Husband	Tall slim dark blue trousers and a top with purple cap
TW	F	45yrs	Mother in-law sister	Tall fair wearing wrapper blue top and head tie with red veil

TIME	ACTOR INITIALS	RECIPIENT INITIALS	WHAT HAPPENED	FOOD	Reactivity
7:40	MM	BB	Mother giving the baby pap with MNP, she told me that the child does not want to eat anything	Liquid cold pap	She was looking at me and forcing the child to take the pap and she says to me he doesn't want to take anything except biscuit
7:45	MM	BB	Mother still forcing the baby to take the pap and he is crying but he is taking it	Liquid cold pap	
7:50	MM	BB	Mother breast feeding the baby	Breast milk	
7:55	MM,TB		Mother and two daughters in-law are pounding millet together in the same motter at the same time		
8:00	MM		Mother washing the grains		
8:05	FI,GS		Father in-law sitting on a stone with his grand son		
8:10	MM	BB	Baby is crying and the mother is breast feeding him		
8:15	BB2		A baby wake up crying, he is suffering from wasting and edema		

TIME	ACTOR INITIALS	RECIPIENT INITIALS	WHAT HAPPENED	FOOD	Reactivity
8:20	MI	BB2	Mother in-law took him give him pap and water and hold him on her lap	Pap	
8:25	MM	BB	Mother breast feeding the baby	Breast milk	
8:30	MM	BB	Breast feeding the baby continue	Breast milk	
8:35	MM	BB	Baby is crying and the mother is giving him water		
8:40	MM		Mother and her husband wife are pounding millet she washed earlier to turn to flour	millet	
	TF,SD		The other two daughters are pounding the remaining millet grain in another motter.		
8:50	SG	BB	Small girl is holding the baby on her lap		
8:55	FI,TF, SD		All of them are still pounding the millet together	Millet	
9:00	BB		Baby is playing		
9:05	TB,MM,TF, SD		The daughters in-law are still processing the millet		
9:15	BB		Baby is playing with his cousin		
9:20	TB,MM,TF SD		The daughter in-laws still processing the millet	Millet	
9:25	FI		Father in-law went out with a hoe, cutlass and a bag		
9:30	MI		Mother in-law is processing the sorghum beating the unprocessed sorghum with a strong stick to remove the grains from the stick	Sorghum	
9:35	BB,SB		Baby is crying, small boy is eating tuwon dawa and dried baobab soup<br>The in-laws finished processing the millet into a paste and keep it on top of the roof in a bowl	Tuwon dawa and baobab soup	
9:40	MM	BB	The small girl took him to his mother, she sat down and breast feed the baby	Breast milk	

TIME	ACTOR INITIALS	RECIPIENT INITIALS	WHAT HAPPENED	FOOD	Reactivity
9:45	TB,MM		The two daughters in-law are sitting and knitting cap with thread and needle		
9:50	SD		The daughter in-law is making a necklace with beads, thread and needle		
	TF MM TB		The other three in-laws are knitting cap with thread, beads and needle		
9:55	MM	BB	Baby is crying and the mother breast feed him	Breast milk	
10:00	MM	BB	Baby still cries, mother gave him pap and he refused, she then breast feed him	Breast milk	
10:05	HI,H2		They came back and took the water that was used to wash the grains and feed the animal		
10:10	SG	BB	Small girl is taking the pap and also giving the baby but he took small and stop taking	Pap	
10:15	BG	BB	The small girl (daughter)is sitting on the ground with the baby playing		
10:20	H1,H2		The two husband are giving the animals (goats and sheep) dried chaff of grain in a calabash after they finished they brought fresh grasses and drop it in front of them		
10:25	BB2		The emaciated baby is drinking pap, he started passing stool (diarrhea) his grandmother washes his body and pack the stool		
10:30	BB, TF, TB MM, SD		Baby is playing on the ground The in-laws are still knitting cap and necklace		
10:35	BB2		The emaciated baby is passing stool (diarrhea)		
10:40	MM	BB	Baby is crying and the mother took him and breast feed him	Breast milk	
10:45	MM	BB	Breast feeding continues	Breast milk	
10:50	MM	BB	Breast feeding continues	Breast	

TIME	ACTOR INITIALS	RECIPIENT INITIALS	WHAT HAPPENED	FOOD	Reactivity
				milk	
10:55	M1,H1,H2		Mother in-law chatting with her children together with a the emaciated baby		
11:00	MM	BB	Mother went to the room with the baby		
11:05	MM	BB	Mother breast feeding the baby lying down on the bed	Breast milk	
11:10	BB		Baby is sleeping		
11:20	BB		Baby is sleeping		
11:25	BB		Baby is sleeping		
11:30	MM		Mother is knitting		
11:35	BB		Baby is sleeping		
11:40	BB		Baby is sleeping		
11:45	MM	BB	Mother and the baby are sleeping		
11:50	BB		Baby is sleeping		
11:55	BB2		The emaciated baby is always crying		
12:00	BB		Baby is sleeping		
12:05	MM	BB	Baby woke up and the mother breast feed him	Breast milk	
12:10	MM	BB	Mother feeding the baby	Breast milk	
12:15	MM	BB	Mother is still breast feeding the baby	Breast milk	
12:20	MM	BB	Baby is still crying and the mother gave him water	Water	
12:25	MM	BB	Mother breast feeding the baby	Breast milk	
12:30	MM	BB	Baby starts crying and the mother gave him the remaining water		
12:35	MM	BB	Mother went out and took a chair puts the baby on her feet with his legs open and he start		

TIME	ACTOR INITIALS	RECIPIENT INITIALS	WHAT HAPPENED	FOOD	Reactivity
			stooling		
12:40	MM		Mother kept him on the ground fetch water in a kettle and wash his body and pack the stool		
12:45	BB		The baby is playing on his mother lap		
12:50	MM		Mother is knitting cap and the baby is sitting on the ground playing		
12:55	BB		Baby took leaves from the ground and eats		
1:00	MM	BB	Mother breast feeding the baby	Breast milk	
1:05	MM	BB	Mother gave the baby water from the kettle	Water	
1:10	MM		Mother knitting a cap with a baby on her lap		
1:15	M1		Mother in-law washing clothes		
1:20	MM	BB	Mother breast feeding the baby	Breast milk	
I:25	MM	BB	Mother bathing the baby he starts crying, she takes the baby to his grandmother, fetches water in a wide rubber and put the baby inside, the mother gives him the water in the rubber with her hand to drink. Mother starts bathing the baby with sponge and soap		
I:30	MM	BB	Mother breast feeding the baby	Breast milk	
1:35	MM	BB	Mother breast feeding the baby	Breast milk	
1:40	SB,SG		The boy is drinking pap and the small girl is eating tuwon dawa and dried baobab soup leaves	Pap, tuwon dawa and soup	
1:45	MM	BB	Mother gives the baby a black fruits (dunya)	Black fruit (dunya)	
1:50	MM	BB	Baby starts crying and the mother breast feed him		

TIME	ACTOR INITIALS	RECIPIENT INITIALS	WHAT HAPPENED	FOOD	Reactivity
1:55	SD	BB	The youngest daughter in-law took him and sat with him under the tree. She is playing with the baby and giving him pap with a spoon he takes small and stop	Cold pap	
2:05	MM		Mother knitting the cap and the baby is playing		
2:10	SG	BB	Baby playing with his sister		
2:15	SG	BB	Sister gives the baby water	Water	
2:20	MM	BB	Baby starts crying and the mother breast feed him	Breast milk	
2:25	MM	BB	Mother breast feeding the baby	Breast milk	
2:30	MM	BB	Mother is backing the baby, she carries the kettle and enter the toilet		
2:35	MM		Mother keeps the baby on the bed, change her wrapper and pray. The baby start crying		
2:40	MM	BB	Mother breast feeding the baby	Breast milk	
2:45	BB		Baby starts crying and the mother gave him water		
2:50	BB		Baby is still crying		
2:55	MM	BB	Mother breast feeding the baby while lying down on the bed	Breast milk	
3:00	MM	BB	Mother is breast feeding the baby	Breast milk	
3:05	BB		Baby is sleeping		
3:10	M1	BB2	Emaciated baby is crying and the mother in-law gave him pap	Cold pap	
3:15	FF,MM,TB, SD		One of the daughter in-law brought out sorghum in a calabash, she divides it into two, and they started pounding the half and the other two		

TIME	ACTOR INITIALS	RECIPIENT INITIALS	WHAT HAPPENED	FOOD	Reactivity
			daughter in-laws take the remaining and start pounding.		
3:25	TW,BB		A woman came to the house with sorghum in a calabash they greeted her, collect the sorghum and divide into two and starts pounding (I was told that she is a sister to the mother in-law, baby still sleeping		
3:30	SG		The small girl is washing her clothes		
3:35	MM,TF,TB SD		The daughter in-law are sitting under the tree on a local bed, baby still sleeping		
3:40	M1		The mother in-law is sleeping with the emaciated baby		
3:45	TF, MM, SD, TB		All the daughters in-law are chatting		
3:50			All the daughter in-law carry bucket and went out to fetch water		
3:55	M1	BB2	Emaciated baby wakes up crying, the mother in-law gave him water and pap, he just took small and starts crying again	Water and pap	
4:00	BB		Baby is still sleeping		
4:05	SB		The small boy comes back home and the mother in-law gave him pap	Pap	
4:10	BB		Baby wakes up and start crying		
	SG	BB	Small girl took him and gave him water	water	
4:15	M1	BB	The baby is still crying and the mother gave him pap, he took small and continues crying		
4:20	MM	BB	Mother breast feeding the baby	Breast milk	
4:25	TW		The woman is removing the chaff from the grains using calabash without adding water		
	MM		Mother is washing the grains,		

TIME	ACTOR INITIALS	RECIPIENT INITIALS	WHAT HAPPENED	FOOD	Reactivity
4:30			the baby is with the youngest daughter in-law		
4:35	SD	BB	The youngest daughter in-law is giving the baby pap, he is crying and refusing to take the pap but taking it small he is crying, she gives him water	Pap	
4:40	BB		Baby is playing with his cousin sister and brother		
4:45	MM	BB	Mother breast feeding the baby after she backs him		
4:50	TB		One of the daughter in-law and the children went outside and climb the tree to get the leaves while am sitting in the house watching them		
4:55	TW		The mother in-law sister put her grains in her bag and ties it and also the chaff in another bag, dried kenaf (rama) and she left. She said to me I just come to see my sister		
5:00	M1		Mother in-law left with the grains in a calabash covered and went to the nearby village for grinding		
5:05	TB,MM,SB SD		They came back with moringa, sat down and started removing the leaves from the stem		
5:10	MM	BB	Mother breast feeding the baby	Breast milk	
5:15	MM	BB	Breast feeding the bay	Breast milk	
5:20	TF		One of the daughter in-law is washing her clothes		
5:25	MI		Mother in-law came back		
	HI		One of the husband came back with bread and mother in-law took and distribute to children and kept some.		
5:30	MM,TB		The two daughter in-laws are sieving the sorghum flour		
	TD		One of the daughter in-law is		

TIME	ACTOR INITIALS	RECIPIENT INITIALS	WHAT HAPPENED	FOOD	Reactivity
			setting the fire		
	TF	BB	Baby starts crying and one of the daughter in-law gives him water, he continues crying		
5:35	MM	BB	She took the baby to his mother and sat down and breast feed him	Breast milk	
	MM	BB	Mother backing the baby		
5:40	MM,TB		One of the daughter in-law goes to the mother in-law room and fetch one bowl full of sorghum and they put pour it into a motter and starts pounding	Sorghum	
	MM	BB	Baby is sleeping on his mother back		
5:45	TF,TD		The fire is burning and one of the daughter in-law is washing the pot, the other fetch water from the bucket and pour into the pot		
	BB2		Baby (emaciated) wakes up and starts crying. The small girl (cousin sister) took him and gave him water, the boy is still crying, she backs him and went out with him		
5:50	BB		Baby is sleeping		
5:55	F1		Father in-law just came back, he fetches sorghum chaff from a bag and gave the animals	Sorghum	
		BB	Baby wakes up crying on his mother back, she brought him down, sit and breast fed him	Breast milk	

Primary Questions (Complete after you complete data collection):

From what you have seen observing THIS HOUSEHOLD, please answer the following questions in detail:

Question	Write your detailed response here
1. In this household, what would be the best way(s) to give nutritional supplements such as an MNP to young children to ensure compliance?<br><br>Why?	Through the mother because she takes care of the children and they are living with the children always. Their father during the dry season as I was told leave the house and travel to a place where there is grass with their cows until raining seasons starts. I was told that they are now in Kantogora leaving their wives, children, mother and their younger brothers to take care of the family until their return.<br>Mother in-law has a role to play because there are some little children that their mother left with the father, she is the one taking care of the children. Also the daughters in-law helps one another in taking of the children.
2. In this household, who is favored during meal preparation or feeding?<br><br>How is this favoritism expressed?	The younger ones in the house, especially the baby whatever comes into the house, they give the children first, baby doesn't want to eat anything, the mother is always breast feeding him
3. How could we promote the MNP supplement for this family that would attract their attention?<br><br>Who would they listen to?	Through the village head, because they accept whatever comes from him and obeys his instructions. he is always with them even if their husband are not around he can decides on their family
4. In this household, what would be the best ways to promote the MNP but limit how much it would be shared?<br><br>Why?	Through proper sensitization to the mothers about the MNP because they are the care givers, they will be the ones to administer the MNP and also make sure that the child takes the MNP alone without sharing it with another child
5. What other issues and concerns did you see with this family that are important for us to consider with regard to introduction of an MNP?	Lack of adequate food, it is only pap (kunu) , fura that is available and the children does not want to take the pap. They only prepared Tuwo in the evening as dinner.<br>Moreover, in this village animals are taken care of more than the people in the house. Their animals are looking very heathy and well fed even the chickens in the house is are looking very healthy

Why?	while people in the house are looking malnourished.
6. Based on your experience observing this child today, do you believe reactivity negatively impacted your ability to see the food-related behaviors of the child?<br><br>Please explain your answer in detail.	No, because when I entered the house I saw the mother trying to force the baby to take the pap and he is refusing and crying. She told me that she added the medicine that was brought to them, that is why she wants him to take even if it is small, besides this, everything went on normal
